# Molecular dissection of Janus kinases as drug targets for inflammatory diseases

**DOI:** 10.3389/fimmu.2022.1075192

**Published:** 2022-12-08

**Authors:** Sunghark Kwon

**Affiliations:** Department of Biotechnology, Konkuk University, Chungju, Chungbuk, Republic of Korea

**Keywords:** janus kinase, signal transducer and activator of transcription, cytokine, autoimmune disorder, kinase inhibitor

## Abstract

The Janus kinase (JAK) family enzymes are non-receptor tyrosine kinases that phosphorylate cytokine receptors and signal transducer and activator of transcription (STAT) proteins in the JAK-STAT signaling pathway. Considering that JAK-STAT signal transduction is initiated by the binding of ligands, such as cytokines to their receptors, dysfunctional JAKs in the JAK-STAT pathway can lead to severe immune system-related diseases, including autoimmune disorders. Therefore, JAKs are attractive drug targets to develop therapies that block abnormal JAK-STAT signaling. To date, various JAK inhibitors have been developed to block cytokine-triggered signaling pathways. However, kinase inhibitors have intrinsic limitations to drug selectivity. Moreover, resistance to the developed JAK inhibitors constitutes a recently emerging issue owing to the occurrence of drug-resistant mutations. In this review, we discuss the role of JAKs in the JAK-STAT signaling pathway and analyze the structures of JAKs, along with their conformational changes for catalysis. In addition, the entire structure of the murine JAK1 elucidated recently provides information on an interaction mode for dimerization. Based on updated structural information on JAKs, we also discuss strategies for disrupting the dimerization of JAKs to develop novel JAK inhibitors.

## Introduction

The Janus kinase (JAK)-signal transducer and activator of transcription (STAT) signaling pathway is involved in various cellular phenomena, such as cell division, apoptosis, inflammatory reactions, and carcinogenesis (1–8). The initial reaction in the JAK-STAT signaling pathway is triggered by binding of extracellular ligands, such as cytokines, to transmembrane type I and II cytokine receptors, which causes receptor dimerization. This multimeric state induces access of JAKs to the dimeric receptor, resulting in autophosphorylation of JAKs. Activated JAKs add phosphate groups to their receptors, enabling STATs to recognize the phosphorylated receptors. The binding of STATs to their receptors facilitates STAT phosphorylation by JAKs. These activated STATs form dimers and translocate to the cell nucleus. Finally, STATs bind to specific DNA regions, causing transcription of target genes. Because these target genes are associated with many cellular processes, including immunity, the JAK-STAT signaling pathway plays a vital role in immune response (2, 6–8).

JAK proteins are non-receptor tyrosine kinases that are classified into four groups: JAK1, JAK2, JAK3, and tyrosine kinase2 (TYK2) (9, 10). While JAK1, JAK2, and TYK2 are produced in most cell types, JAK3 is produced only in hematopoietic and lymphoid cells (11). Regardless of JAK isoforms, however, JAKs are responsible for immune responses including interferon signaling (12, 13). To perform their biological functions, JAKs form homodimers or heterodimers by binding to the same isoform or other forms. The binary combination of JAKs differs depending on specific receptors that bind their own ligands (7, 14). Each dimeric JAK is involved in specific biological functions, most of which correspond to immune response (7, 14). Accordingly, dysfunctional JAKs can cause severe immune disorders by precluding normal downstream signaling in the JAK-STAT pathway.

Owing to their importance in the JAK-STAT pathway, JAKs have been attractive drug targets to develop inhibitors that block cytokine signaling. Starting with ruxolitinib approved in 2011 (15, 16), numerous JAK inhibitors have been approved and launched in the global pharmaceutical market (17–32). They target one or more than one kinases among JAK1, JAK2, JAK3, and TYK2, accordingly aiming at different indications such as myelofibrosis, rheumatoid arthritis, atopic dermatitis, and psoriasis (13, 14, 33). Several review papers provide valuable information on JAKs and their inhibitors in the treatment of specific indications. Roskoski Jr. introduced JAK inhibitors with a focus on the treatment of neoplastic and inflammatory disorders, along with depiction of structural features of JAKs (13). Huang et al. summarized JAK inhibitors in clinical trials of COVID-19 (14). Inhibition of JAK activities constitutes an effective treatment strategy, in that JAKs are key molecules associated with upstream signal transduction in the JAK-STAT pathway. They also have diverse isoforms and dimeric combinations, depending on specific ligands, including cytokines. In particular, JAK3 can be a suitable drug target to treat adaptive immune disorders, considering that JAK3 kinases are produced in specific cells such as hematopoietic cells and lymphocytes (11).

Although various inhibitors targeting JAKs have been approved and launched, JAK inhibitors have two intrinsic limitations as therapeutic agents, i.e., a lack of selectivity and potential drug resistance. These issues have commonly been raised with other kinase inhibitors (34, 35). Considering that all kinases require an ATP molecule as one of their two substrates, assuming that spatial features of the ATP-binding site are shared to some extent among kinases is reasonable. Indeed, approved JAK inhibitors competitively bind to the ATP-binding site (36–39), which signifies that JAK inhibitors can target not only JAKs but also other kinases. Moreover, mutations in *JAK* genes can create structurally altered JAK proteins, leading to drug resistance to JAK inhibitors by weakening the affinity of JAK inhibitors. Therefore, understanding the mode of inhibition of JAK inhibitors and structural features of JAKs is indispensable for developing novel and specific JAK inhibitors. In addition, the entire structure of murine JAK1 was elucidated recently. This structure showed a unique dimerization mode of JAK1. This structural information can lead to a new approach to the development of JAK inhibitors.

In this review, we describe previously known structures of JAKs to obtain structural insight into their inhibitory mechanisms. We also discuss conformational changes of JAKs for catalysis. Based on updated structural information on JAKs including the entire structure of murine JAK1, the potential inhibitor-binding sites of JAKs are explored to develop novel and specific inhibitors. This review enables us to profoundly understand the molecular biology of JAKs, which can ultimately lead to the development of novel anti-inflammatory agents.

## Architecture of JAKs

In general, JAKs consist of seven JAK homology (JH1-7) domains from the C terminus to N terminus (2, 40, 41). The JH1 and JH2 domains correspond to kinase and pseudokinase domains, respectively. The JH3-5 domains form an Src homology 2 (SH2) domain, and the JH6-7 domains correspond to a band-4.1 protein, ezrin, radixin, and moesin (FERM) domain. The SH2 and FERM domains are directly involved in the binding of JAKs to cytokine receptors.

**Figure 1:**
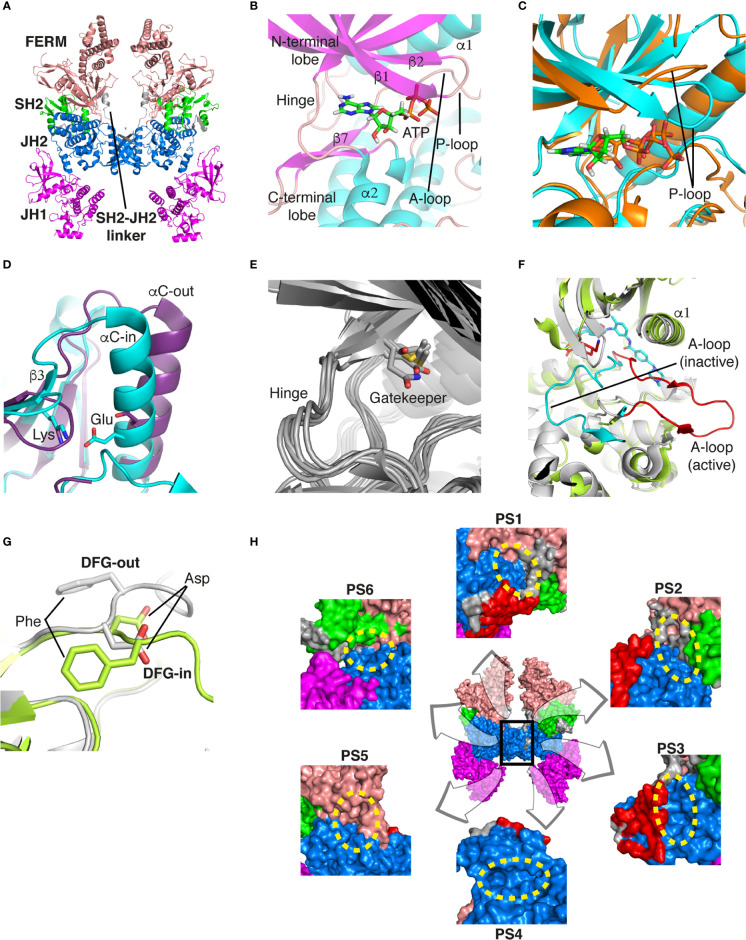
Structural analysis of JAK. **(A)** The entire structure of murine JAK1 as a dimeric form (PDB ID: 7T6F) is represented as cartoon. IFNΛR is omitted for clarity. The JH1 (magenta), JH2 (marine), SH2 (green), and FERM (salmon) domains are shown. The SH2-JH2 linker is located between the JH2 and SH2 domains. **(B)** The ATP-binding site of the JH2 structure of JAK2 (PDB ID: 5I4N). The JH2 domain is divided into the N- and C-terminal lobes. The ATP molecule is depicted as sticks. **(C)** Different conformations of the P-loop. The ATP-bound form (cyan; PDB ID: 5I4N) is superimposed onto the ADP-bound form (brown; PDB ID: 4GVJ). The adenosine moiety of ATP and ADP is colored green and gray, respectively. **(D)** Two conformations of the C-helix. The αC-in state form (cyan; PDB ID: 5CSW) is superimposed onto the αC-out state form (violet; PDB ID: 5L3A). **(E)** Gatekeeper residues. The four JH1 (PDB ID: 6HZU, 6X8G, 7Q6H, and 7REE) and three JH2 (PDB ID: 4L01, 7AX4, and 7JYQ) domain structures are superimposed onto each other. Met and the other residues are colored yellow and gray, respectively. **(F)** Two conformations of the A-loop: active (red) and inactive (cyan) conformations. The structure of the JH1 domain of JAK1 (limon; PDB ID: 6HZU) is superimposed onto that of an ABL kinase (gray; PDB ID: 1IEP). The A-loops of JAK1 and ABL are colored red and cyan, respectively. Imatinib is represented as sticks. **(G)** Two conformations of the DFG-motif. The DFG-in form (limon; PDB ID: 1IEP) is superimposed onto the DFG-out form (gray; PDB ID: 5L3A). **(H)** Potential inhibitor-binding sites of JAKs. The entire structure of murine JAK1 as a dimeric form is shown in the center, and represented as surface. The structure comprises the FERM, SH2, SH2-JH2 linker, JH2, and JH1 domains. The interface for dimerization is colored red. The other color code is the same as that in the panel **(A)**. The six yellow dashed ellipses indicate each potential inhibitor-binding site, which the letters ‘PS’ denote.

Recently, the entire structure of murine JAK1 in complex with part of the interferon lambda receptor (IFNλR) was determined as a homodimeric form using cryo-electron microscopy (PDB ID: 7T6F) (**Figure 1A**) (42). Although JAK1 was known to form a heterodimer with other types of JAKs such as JAK2, JAK3, and TYK2 in the cellular environment (14, 43), this dimeric structure provides valuable information on the entire architecture of JAK1 as a snapshot of its active form.

Of the seven domains of JAKs, the JH1 and JH2 domains are directly associated with catalytic function as kinases, although they exhibit different catalytic activities (10, 44, 45). These two domains are structurally similar, along with similarities in amino acid sequences (46). Since all four JAK family members (JAK1-3 and TYK2) are kinases, they share structural features common with those of all kinases. The structure of the JH2 domain of JAK2 (PDB ID: 5I4N) (47) containing an ATP molecule (one of its two substrates) at the active site provides structural information on the ATP-binding mode. The JH2 domain consists of two parts: a small N-terminal lobe and a large C-terminal lobe. The N-terminal lobe is linked to the C-terminal lobe by a hinge region (**Figure 1B**). These two lobes create a cleft in the linkage region, which constitutes the active site (**Figure 1B**).

An ATP molecule binds to the active site, which is surrounded by a β-sheet in the N-terminal lobe and the α2 helix in the C-terminal lobe (**Figure 1B**). A loop connecting the β1 and β2 strands is located next to the α1 helix (**Figure 1B**). This loop is known as P-loop or G-loop (i.e., a Gly-rich loop). The α1 helix in the N-terminal lobe is called the C-helix (αC) (**Figure 1B**). The β7 strand in the C-terminal lobe is linked to a relatively long loop (**Figure 1B**). This loop is called A-loop (i.e., an activation loop). The A-loop can adopt different conformations, including a helical form, in response to substrates binding to the active site.

## Conformational changes of JAKs for catalysis

As other kinases have several flexible regions in the proximity of active sites, the active sites of JAKs are also surrounded by several loops along with the C-helix, which undergo conformational changes in response to their substrates or inhibitors. The P-loop is directly associated with positioning of the phosphate groups of ATP. Specifically, the Gly554, Thr555, and Thr557 residues in the P-loop form hydrogen bonds with phosphates. However, owing to the substantial flexibility of this loop, this region appears disordered in several crystal structures. Compared with the ATP-bound form (PDB ID: 5I4N) (47), the P-loop opens outwards in the ADP-bound form (PDB ID: 4GVJ) (48) (**Figure 1C**). Namely, the P-loop residues do not interact with ADP as tightly as that with ATP. This structural difference seems reasonable, in that ADP, as a product of catalysis needs to be released from the active site.

The C-helix also plays an important role in JAK catalysis. A Lys residue of the conserved AXK motif (X = any amino acid) in the β3 strand can form a salt bridge with a Glu residue in the C-helix at an appropriate position (αC-in state) (**Figure 1D**; cyan). The Lys residue is a key residue in JAK catalysis, which corresponds to K908 (JAK1), K882 (JAK2), K855 (JAK3), and K930 (TYK2). However, spatial distortion of the C-helix gives rise to a deviation from the C-helix-in state, thereby disrupting the salt bridge (αC-out state) (**Figure 1D**; violet). Therefore, the C-helix-in state facilitates catalysis by rendering correct position of the Lys residue.

A residue in the β5 strand adjacent to the hinge region is called ‘gatekeeper’. The gatekeeper plays a vital role in controlling the access of ATP or inhibitors to the hydrophobic backpocket. This residue varies depending on the type and domain of JAKs. Specifically, JH1 domains of all four JAKs (JAK1, JAK2, JAK3, and TYK2) retain a Met residue as the gatekeeper (**Figure 1E**). In contrast, Glu (JAK1), Gln (JAK2), and Thr (TYK2) residues are assigned to the same position in JH2 domains (**Figure 1E**). This gatekeeper is critical for sensitivity to inhibitors. Mutation of the gatekeeper to other residues, such as bulkier residues, can decrease the affinity of inhibitors by causing steric hindrance to inhibitor binding (49).

Remarkably, the A-loop shows two distinct conformations. In the ATP-bound form, the A-loop is located distantly from the P-loop (active conformation) (**Figure 1F**). This conformation renders the active site constructed, thereby facilitating the binding of ATP and its target protein substrate to the active site. Because this conformation provides the architecture of the ATP-binding pocket, numerous inhibitors have been developed to bind to the ATP-binding site in this active conformation (49). In contrast, in the “rest period” of kinases, the A-loop exhibits a form moved towards the P-loop (inactive conformation) (**Figure 1F**). However, JAK structures in the inactive conformation have not yet been determined. The inactive conformation of Abl, a tyrosine-protein kinase (PDB ID: 1IEP) (50), is described in this review (**Figure 1F**). This conformation does not form an ATP-binding pocket. Alternatively, this inactive conformation renders the space between the A-loop and C-helix wider, which implies that this region can be another target site for inhibitors. Indeed, the structure of Abl shown here (PDB ID: 1IEP) contains imatinib as an inhibitor in the inactive conformation (50) (**Figure 1F**). Consequently, the conformational change in the A-loop is the most striking structural phenomenon concerning kinase catalysis, which is noteworthy in that this structural information can lead to the development of novel JAK inhibitors.

The conserved DFG-motif is another significant part of the conformational changes involved in catalysis (49). This motif is located at the N terminus of the A-loop. In the active conformation, the Asp residue of the DFG-motif orients inward the ATP-binding site and coordinates with a Mg ion (DFG-in) (**Figure 1G**; gray). In the inactive conformation, the Asp residue orients outwards from the ATP-binding site, where it cannot coordinate with a Mg ion in the ATP-binding site (DFG-out) (**Figure 1G**; limon). Therefore, the DFG-motif regulates the catalytic activity of kinases by suitably adopting their respective conformations.

## Development strategies for novel JAK inhibitors

In general, kinase inhibitors are classified into six types depending on their binding sites and kinase conformations (types I-VI) (51). Type I inhibitors bind to the ATP binding site in the active conformation (DFG-in and αC-in). Type I^1/2^ inhibitors bind to the same site in an incomplete and inactive conformation (DFG-in and αC-out). Type II inhibitors bind in the inactive conformation (DFG-out and αC-out). In contrast to types I and II, the binding site of type III inhibitors is located near the ATP binding site. Type IV inhibitors bind to an allosteric site far from the ATP binding site. Accordingly, type III and IV inhibitors target allosteric sites, in that they do not bind to the ATP binding site. Type V inhibitors bind to the ATP-binding or allosteric site. Lastly, type VI inhibitors covalently bind to the ATP binding or allosteric site.

To date, all JAK inhibitors launched into the pharmaceutical market are type I inhibitors, except for inhibitors unidentified in the PDB (13). Information on launched JAK drugs is summarized in **Table 1**. However, type I inhibitors compete with ATP for the same binding site. This signifies that type I inhibitors can bind to other unintended kinases which have ATP-binding sites. Nonspecific binding usually results in unexpected side effects. Therefore, discovering target sites other than the ATP-binding site is of recent interest in developing new inhibitors for a specific kinase. Accordingly, although the current JAK drugs are type I inhibitors, other types of JAK inhibitors can be designed and developed to enhance JAK-binding specificity.

**Table 1:** Human JAK inhibitor profiles^a^.

Generic name	Brand name	Target	Type	First Approval year	Approval country	PDB ID^b^	Chemical structure
Ruxolitinib	Jakafi/Jakavi	JAK1, JAK2, JAK3, TYK2	I	2011	US	6VGL	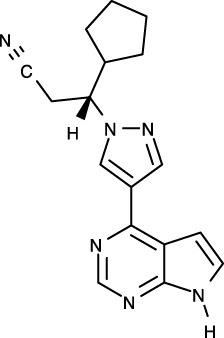
Tofacitinib	Xeljanz	JAK1, JAK2, JAK3, TYK	I	2012	US	3EYG, 3FUP, 3LXK, 3LXN	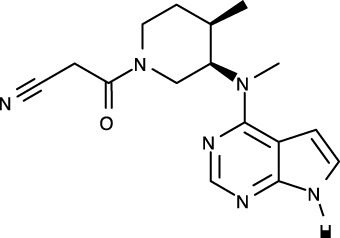
Baricitinib	Olumiant	JAK1, JAK2, TYK2	I	2017/2022	EU/US	6WTO	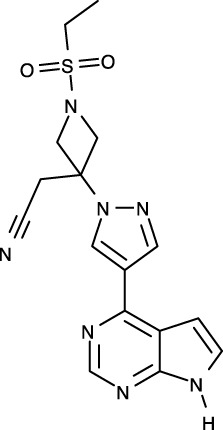
Peficitinib	Smyraf	JAK1, JAK2, JAK3, TYK2	I	2019	Japan	6AAH, 6AAJ, 6AAK, 6AAM	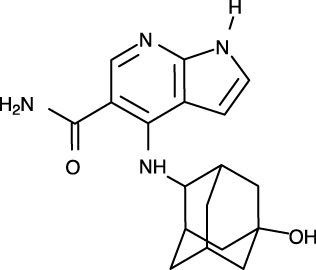
Fedratinib	Inrebic	JAK2	I	2019	US	6VNE	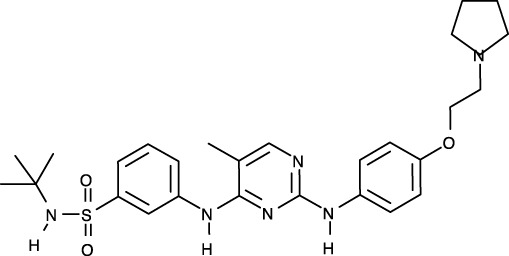
Upadacitinib	Rinvoq	JAK1	–	2019	US	–	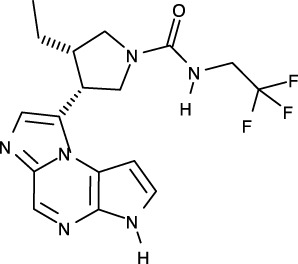
Filgotinib	Jyseleca	JAK1	I	2020	US/Japan	4P7E, 5UT5	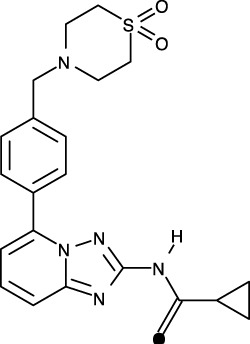
Delgocitinib	Corectim	JAK1, JAK2, JAK3, TYK2	I	2021	Japan	7C3N	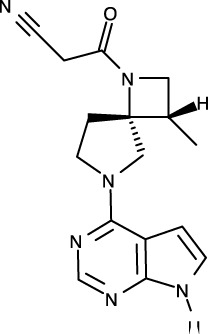
Abrocitinib	Cibinqo	JAK1	I	2021/2022	EU/US	6BBU, 6BBV	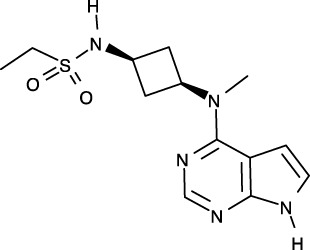
Pacritinib	Vonjo	JAK2	–	2022	US	–	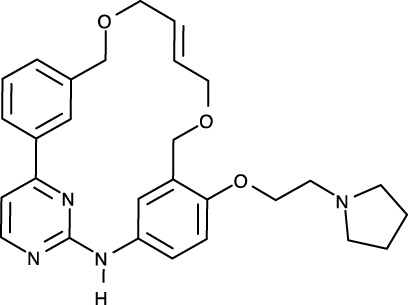

^a^ Ref. (13, 14, 33).

^b^ Inhibitor-complex structure.

Notably, two monomeric JAKs also form a dimer when two monomeric cytokine receptors form a dimer upon binding a cytokine (13, 14, 43). The resulting JAK dimer facilitates the *trans*-phosphorylation of its counterpart (13, 14, 43). Dimerization of JAKs is a prerequisite for JAK-STAT signaling transfer; therefore disturbing JAK dimerization may constitute a novel strategy for blocking the JAK-STAT signaling pathway at the upstream level. Hubbard proposed a putative mechanism for JAK2 activation (52). In this review, the author explained JAK2 activation with vast conformational changes. According to this hypothesis, monomeric JAK2 exists in the inactive conformation in the equilibrium state (52). Namely, the JH1 domain is attached to the JH2 domain, thereby maintaining the autoinhibited state. When a cytokine binds to its receptor, dimerization of the receptor by the cytokine induces JAK2 dimerization. V617F, a JAK2 mutant in the JH2 domain, which causes myeloproliferative neoplasms, has been previously identified (53–56). This mutant may induce a substantial conformational change in the JH1 domain, resulting in the active conformation of JAK2, despite the absence of a cytokine (52). Moreover, the V617F mutant may reinforce the interface between monomers owing to the bulkier side chain. Consequently, the V617F mutant leads to abnormal JAK-STAT signaling by maintaining its active dimeric form without external stimuli.

The V617 residue of human JAK2 corresponds to the V660 residue of the murine JAK1, which is associated with the dimerization of murine JAK1. The substitution of V660 with Phe presumably increases the hydrophobic interaction between the two Phe residues through π- π stacking. However, determining the extent to which this hydrophobic interaction affects the dimerization is challenging.

As JAK dimerization is a critical step for the *trans*-phosphorylation of the JH1 domain, blocking the dimerization of JAKs may constitute a new paradigm for developing JAK inhibitors. The entire structure of JAK1 (PDB ID: 7T6F) (42) provides structural insight into potential inhibitor-binding sites to block dimerization. However, this structure is that of murine JAK1, not human JAK. Several clefts (PS1-6), which could be novel inhibitor-binding sites, were identified between the JH2 domain and adjacent domains (**Figure 1H**). The PS1 and PS2 clefts comprise part of the FERM, SH2-JH2 linker, and JH2 domains, whereas the PS3 cleft consists of part of SH2-JH2 linker and JH2. The PS4 cleft corresponds to the ATP-binding site of the JH2 domain. The PS5 cleft, which comprises part of the FERM and JH2 domains, is located at the rear of the JH2 interface. Lastly, the PS6 cleft consists of part of FERM, SH2, SH2-JH2 linker, and JH2. These clefts appear relatively large and sufficiently deep to accept small molecules. Furthermore, all clefts except PS3 are located at the interface of the domains. This implies that the binding of suitable small compounds to the PS clefts might trigger a conformational change in the JH2 domain, thereby affecting and obstructing its dimerization. However, whether these sites induce significant conformational changes remains to be determined.

## Discussion

To date, several JAK inhibitors have been approved and launched in the pharmaceutical market. Structures of the JAK-inhibitor complexes have also been reported (36–39). Such structural information provides insight into the inhibitor-binding modes. However, considering that the affinity of many kinase inhibitors has decreased owing to the occurrence of drug resistance (49), the inhibitory ability of the JAK drugs may also reduce eventually.

The mutation of key residues in the active site of kinases is one of the most prevalent molecular mechanisms underlying inhibitor resistance (49). One of the most common mutation sites is the gatekeeper residue. The size and shape of the gatekeeper residue regulate the access of a molecule binding to the hydrophobic back pocket; therefore mutations in the gatekeeper residue can reduce the affinity of kinase inhibitors to the ATP-binding site. Gatekeeper mutations have also been identified in several kinases (57–60). The A-loop is another mutation site for kinase inhibitor resistance (49). Mutations in the A-loop are more variable than those in gatekeeper mutations. Such mutations affect the binding of inhibitors to kinases in the inactive conformation of the A-loop (49). Mutations in the A-loop typically lower the affinity of the inhibitors for the inhibitor-binding site in the inactive conformation by maintaining the active conformation of the A-loop. Therefore, mutations in the A-loop appear to be critically related to the conformation.

Natural JAK-related mutations associated with inhibitor resistance in patients have not been reported. However, a recent study indicated that several mutations in JAK2 induce resistance to ruxolitinib at the cellular level (61). In this study, the authors reported that the Y931C, L983F, and G993A mutants of murine JAK2 cause acquired resistance to ruxolitinib (61). These residues were associated with the ATP/ruxolitinib binding site. Although these residues may not constitute naturally occuring mutation sites for resistance to ruxolitinib, these *in vitro* results may provide valuable information for developing novel JAK2-inhibitors.

The entire structure of murine JAK1, which was recently elucidated (42), helped us understand the detailed molecular mechanisms of its receptor binding and dimerization. Notably, the dimerization mode of the JH2 domain suggests novel strategies for JAK-inhibitor development. In the JAK-STAT signaling pathway, diverse heterodimers of JAKs except for the JAK2 homodimer are associated with downstream signaling. These combinations include JAK1-JAK2, JAK1-JAK3, and JAK1-TYK2. Although these heterodimeric structures have not been determined, the respective JH2 domains are probably involved in dimerization. If each dimerization mode is the same as in the murine JAK1, the dimerization mode of murine JAK1 may be generalized to all JAK heterodimers. This assumption means that strategies for disrupting dimerization can be applied to these heterodimers. However, the potential inhibitor-binding sites discussed in the previous section are based on the premise that such binding induces significant conformational changes in the JH2 domain to destabilize dimerization. Therefore, future studies should focus on the discovery of potential inhibitor-binding sites and factors that affect conformational changes to obstruct dimerization.

## Author contributions

The author confirms being the sole contributor of this work and has approved it for publication.
